# Acute Renal Infarction Presenting with Acute Abdominal Pain Secondary to Newly Discovered Atrial Fibrillation: A Case Report and Literature Review

**DOI:** 10.1155/2014/981409

**Published:** 2014-12-29

**Authors:** Sherif Ali Eltawansy, Shil Patel, Mana Rao, Samaa Hassanien, Mihir Maniar

**Affiliations:** ^1^Department of Internal Medicine, Monmouth Medical Center, 300 Second Avenue, Long Branch, NJ 07740, USA; ^2^Department of Medicine, Cairo University Medical School, Cairo 11562, Egypt

## Abstract

We report an 85-year-old female with known history of recurrent diverticulitis presented with abdominal pain. It was believed that the patient again needed to be treated for another diverticulitis and was started on the routine treatment. The initial CT scan of abdomen showed renal infarcts bilaterally that were confirmed by a CT with and without intravenous contrast secondary to unknown cause. An ECG found accidentally that the patient was in atrial fibrillation, which was the attributed factor to the renal infarctions. Subsequently, the patient was started on the appropriate anticoagulation and discharged.

## 1. Background

Acute renal infarction is a rare cause of acute abdominal pain. It has to be expected in the patients with cardiovascular risk factors. Most accurate diagnostic tool is the helical CT scan of abdomen. Once it is diagnosed, preferred therapies are percutaneous endovascular therapy, anticoagulation, or thrombolysis. If the diagnosis is missed, there is an increase in mortality and morbidity as a consequence of declining renal function or even failure. Our case was challenging given that she presented with only acute abdominal pain with no previous history of arrhythmias.

## 2. Learning Objective

We report a case with an acute renal infarction presenting only with abdominal pain. The symptoms were initially thought to have been secondary to a diverticulitis according to the previous history. It was later concluded that the pain was due to multiple bilateral renal infarctions. ECG in abdominal pain should be routine during initial work-up and not performed accidentally after the fact. The ECG in our case was found to be in atrial fibrillation. Although it is known to be one of the most common causes of renal infarction, other vasoocclusive events secondary to atrial fibrillation were not included in her initial differentials.

Treatment options include anticoagulation in cases of associated atrial fibrillation. Thrombectomy and intra-arterial thrombolysis could be used sometimes in cases with some success. Angioplasty with stenting could be used in presence of renal artery dissection. Surgery could be an option in cases with renal infarction following blunt abdominal trauma.

## 3. Case Presentation

An 85-year-old Caucasian female presented to the emergency room (ER) with a three-day history of right lower quadrant and periumbilical abdominal pain. Her primary care physician had prescribed oral levofloxacin 750 mg once daily for seven days, for a presumptive diagnosis of diverticulitis. Since antibiotic did not seem to bring about a change in her clinical status, the patient decided to seek help at the ER. She was known to have diverticulosis, several past episodes of left sided diverticulitis, hypertension, hyperlipidemia, hypothyroidism, esophageal reflux, a hiatal hernia, and chronic obstructive pulmonary disease (COPD). Surgery for the recurrent diverticulitis was not done because of the old age of the patient who preferred to use antibiotics and be on follow-up. On chart review, there was a mention of supraventricular tachycardia but the patient had no recall of its nature and records were not available. She had had a cholecystectomy in 1991 and prior colonoscopies which had revealed extensive diverticulosis throughout her descending and sigmoid colon. Her medication list included omeprazole, levothyroxine, and vitamin D. Of note is the fact that she was not on medications for hypertension, hyperlipidemia, and COPD. She had no known allergies. She had 50 pack-year smoking history but quit 10 years ago. Family history was notable for coronary artery disease in her father who died of an acute myocardial infarction.

**Figure 1:**
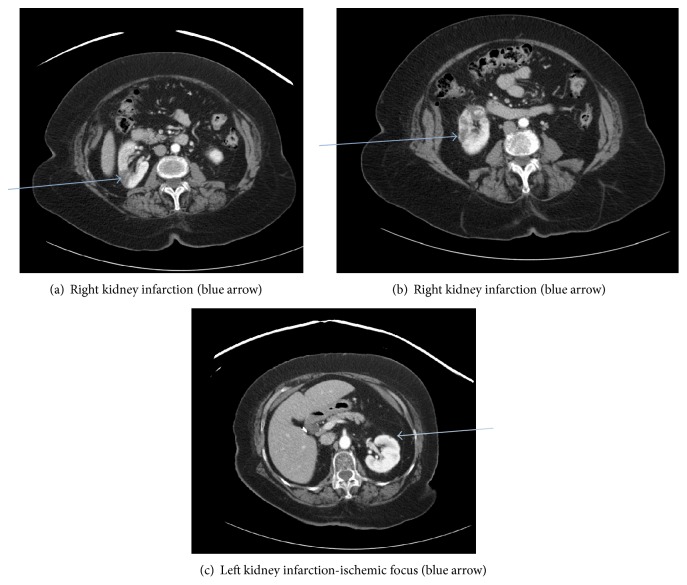
CT abdomen and pelvis with IV contrast. Findings: kidneys: both kidneys were not obstructed. A subcentimeter hypodense lesion is seen without calcification or septation in the upper pole of the left kidney. A wedge-shaped hypodense lesion is seen in the upper pole of the right kidney, which may represent a perfusion abnormality. Additional hypodense regions are seen in the mid pole of the right kidney, which may represent the sequela of a perfusion abnormality. Alternatively, these hypodense regions may represent lesions with soft tissue attenuation. In the lower pole of the right kidney, a 1.6 × 1.8 cm hypodense lesion is seen with internal enhancement.

**Figure 2:**
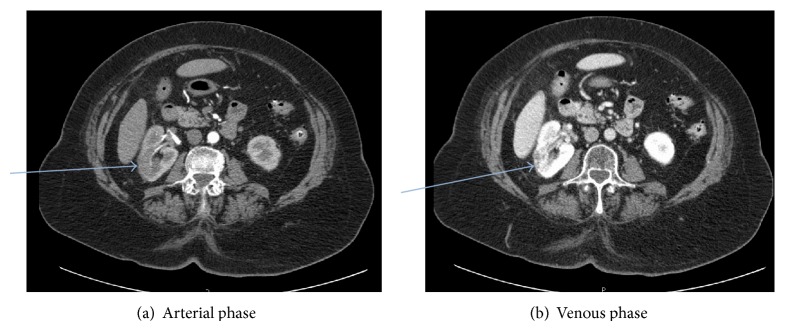
CT abdomen and pelvis with and without contrast (renal protocol). Findings: multiple hypodense foci are present within the right kidney, on all 3 postcontrast series (blue arrows). In addition, there is intraluminal thrombus within the mid to lower pole branch of the right renal artery on the arterial phase. There is normal perfusion to the capsule surrounding these hypodense areas. Therefore, the findings are most consistent with multiple renal infarcts. The previous identified area of concern in the lower pole of the right kidney also likely represents a perfusion abnormality secondary to infarction. There are multiple low-attenuation foci within the kidneys bilaterally which are too small to characterize as well.

On exam, her vital signs were found to be normal. The only significant finding was periumbilical and right lower quadrant abdominal tenderness, without rebound, guarding, or rigidity of the abdomen. Initial testing showed mild leukocytosis with all other laboratories including hemoglobin, platelet count, renal, and kidney chemistries being within the reference ranges. Lipid panel was as follows: total cholesterol 212 mg/dL, triglycerides 167 mg/dL, LDL cholesterol 151 mg/dL, and HDL cholesterol 43 mg/dL. Patient was on diet modification for hyperlipidemia and refused any medications for that. An obstructive series ruled out intestinal obstruction. CRP was not measured as the CT scan results were satisfactory to the managing team. A computed tomographic (CT) scan (Figure 1) of the abdomen with contrast showed diverticulosis coli without surrounding inflammation. Wedge-shaped hypodense lesions were incidentally identified on the upper and mid poles of the right kidney as well as the upper pole of the left kidney. This necessitated a renal protocol CT scan (Figure 2) with and without intravenous contrast, only to confirm multiple infarcts in the right kidney with small ischemic foci in the left kidney. An admission electrocardiogram (EKG) accidentally revealed new onset atrial fibrillation with controlled ventricular response.

Heart rate was still well controlled at 80s and patient was started on oral bisoprolol and apixiban. An echocardiogram was performed and showed ejection fraction of 58.9% with left atrium diameter 2.7 cm. There was moderate to severe tricuspid regurgitation with elevated right ventricular systolic pressures. The patient was discharged to home on apixiban and bisoprolol with marked clinical improvement. Follow-up labs after 3 months were normal showing stable normal kidney function tests. Patient remained in atrial fibrillation with ventricular rate controlled on discharge.

## 4. Discussion

Renal infarctions are rare. It can be missed on patient work-up which makes its documented incidence falsely lower than the true incidence. In a study of 14,411 autopsies published in 1940, the incidence of renal infarction was 1.4 percent [1]. In a later series of almost 250,000 patients seen at an emergency department over four years, only 17 (0.007 percent) were diagnosed with acute renal infarction [2]. The etiology of acute renal infarction is usually due to thromboembolism with the source of emboli arising from the heart or the aorta. The most common disease causing it is atrial fibrillation. An incidence of 2 percent for renal thromboembolism was reported in a series of almost 30,000 patients with atrial fibrillation who were followed up for up to 13 years [3]. This is followed by infective endocarditis, thrombi from suprarenal aorta, renal artery dissection, hypercoagulable status, endovascular intervention, cocaine use, sickle cell disease, or unknown etiology [4–8]. One study retrospectively reviewed 35 cases of segmental renal infarction after nonpenetrating injury in order to assess the clinical significance and the most appropriate management. They were demonstrated by contrast medium-enhanced computed tomography (CT), 19 in the left and 16 in the right kidney. Twenty-five of the thirty-five infarcts (71%) occurred as an isolated renal injury. A distinct upper pole predilection for segmental infarct was observed [9]. Acute renal infarction diagnosis can be missed. Unilateral flank pain in a patient with an increased risk for thromboembolism should raise the suspicion of renal infarction. In such a setting, hematuria, leukocytosis, and an elevated (lactate dehydrogenase) LDH level are strongly supportive of the diagnosis. Korzets et al. had an observational study on patients admitted to a hospital and managed through the emergency department during 36 months including the review of the CT scan of abdomen done during admission. The conclusion was that during the 36-month observation period, the incidence of acute renal infarction was 0.007%. Acute renal infarction is not as rare as previously assumed because it can be missed [10]. The study found that time from admission to the emergency department to definitive diagnosis ranged from 24 hours to 6 days. Obviously, this delay in diagnosis is much too long and points to a lack of physician awareness regarding the entity. This also applies to the radiologist, since in two cases the initial CT interpretation was incorrect. Our overall incidence of 6.1 per million per year probably underestimates the true incidence. Since unenhanced CT is now used almost routinely in the investigation of acute flank pain, it is imperative to remember that contrast enhancement is essential for the diagnosis of acute renal infarction [10].

The clinical presentation of renal infarction can be misleading. The diagnosis of acute renal infarction is often missed or delayed due to both the rarity of the disease and its nonspecific clinical presentation [11, 12]. Bolderman et al. did a study in a single university hospital on 27 patients with idiopathic renal infarctions during a period of 3 years. This included the review of their CT scan. Twenty-five patients (93%) presented with pain that was continuous in all but two and was most often located in the lumbar region. Associated symptoms were nausea in 63% of patients, vomiting in 33% of patients, body temperature in excess of 37.5°C in 41% of patients, and urinary symptoms in 15% of patients. Lumbar tenderness was present in 63% of patients, and abdominal tenderness was present in 74% of patients [13]. Acute elevation of blood pressure can happen and this is explained by the fact that renal infarction can be renin-mediated [14]. Laboratory findings usually include elevated serum lactate dehydrogenase (LDH), C-reactive protein, leukocytosis, microscopic hematuria, proteinuria, elevated serum creatinine, and creatinine kinase. Oliguria can happen [13, 15]. Our case was remarkable only for elevated serum LDH and leukocytosis. In case of diagnosing the renal infarction, exploring the precipitating etiology is the next step. This includes ECG, transthoracic echocardiography, Holter monitoring, thrombophilia panel, homocysteinemia measurement, and magnetic resonance abdominal angiography [15].

In renal infarction patients, the CT angiography is the initial tool of choice, but definite diagnosis is made by renal angiography. The classic finding is of a wedge-shaped zone of peripheral diminished density without enhancement. Conventional ultrasound imaging has been used to evaluate renal infarction, but it can neither diagnose nor exclude acute global renal infarction due to the fact that there is no specific change of the infarcted kidney. In segmental renal infarction, a time-sequence echogenicity change was described in animal experiment, whereas it is nonspecific in humans [16–19]. It is worthy to confirm that conventional ultrasound can be insensitive and that CT scan can even miss the diagnosis and can be misinterpreted as malignant disease for example [20]. Doppler evaluation of renal arterial and venous blood flow should be able to detect global or major segmental renal infarction by demonstrating the absence of blood flow. Yet, segmental renal infarction has more risk to be missed by Doppler than global renal infarction [19, 21, 22]. Hazanov et al. had a case series study on 44 patients with renal infarction with atrial fibrillation. CT scan of the kidney with intravenous contrast media is fast becoming the diagnostic technique of choice for renal embolism. The classic finding is of a wedge-shaped zone of peripheral diminished density without enhancement [23]. A hypoattenuated area with an associated mass effect, which was present in 32% of cases, was followed by the cortical rim sign in 19%. The cortical rim nephrogram sign represents opacification of a rim of functioning nephrons, supplied by capsular collaterals, surrounding an otherwise nonfunctioning kidney [24]. Excretion urography (IVP) or a nuclear renal scan can be used for diagnosis of renal infarction but CT scan with IV contrast remains the best choice [24].

Unenhanced helical CT scan is now thought to be the investigation of choice for the diagnosis of renal colic, since it can be rapidly performed and can detect nearly all types of renal calculi. In addition it may detect extrarenal causes of abdominal pain including appendicitis, diverticulitis, biliary tract disease, leaking aortic aneurysm, and gynecologic disease. However, it cannot easily detect renal artery thromboembolism. Since the clinical picture of renal artery embolism is similar to that of renal colic (flank pain and microscopic hematuria), the widespread use of unenhanced CT scan needs to be reassessed. We suggest that in those patients with clinical characteristics suggesting renal embolus, such as atrial fibrillation without any or without appropriate anticoagulation, unenhanced CT scans of the abdomen should be followed by enhanced scans if no calculi are found [23, 24].

Treatment is clear if the etiology is atrial fibrillation and it will be a conventional anticoagulation with favorable prognosis [25]. In our case, apixiban was used. On follow-up, patient was symptom free with no recurrence of abdominal pain. Laboratory work (including kidney function tests), 3 months after initial presentation, was unremarkable.

There are case reports and case series reporting the use of local intra-arterial thrombolytic therapy and thrombectomy. These studies reported successful reperfusion in most patients without significant therapy-associated complications. However, renal outcomes were only improved in some patients [26–31]. Angioplasty is the used treatment among patients with renal infarction caused by an intrinsic abnormality of renal vessels, such as dissection, angioplasty with stent placement. Surgery can be indicated in selected cases especially post-traumatic renal infarction after a blunt or penetrating trauma [32, 33].

## 5. Conclusion

Renal infarction is a rare cause of acute abdominal pain. Incidence is rare and numbers vary according to different studies (0.007% in [10]). It has to be suspected and managed appropriately, especially in patients with risk factors like cardiac arrhythmias (specifically atrial fibrillation). It has to be on the differential diagnosis of the admitting physician.
